# Postoperative keratitis due to *Paecilomyces*: a rare pediatric case

**DOI:** 10.11604/pamj.2016.24.317.9772

**Published:** 2016-08-18

**Authors:** Ebru Toker, Nihan Ziyade, Serkan Atici, Kepenekli Kadayifçi Eda, Özden Türel, Demet Toprak, Merih Oray, Nilgün Cerikcioglu, Ahmet Soysal, Mustafa Bakir

**Affiliations:** 1 Istanbul University, Istanbul Faculty of Medicine, Department of Ophthalmology, Pendik, Istanbul, Turkey; 2 The Council of Forensic Medicine, Department of Microbiology, Istanbul, Turkey; 3 Marmara University Medical Faculty, Department of Pediatric Infectious Diseases, Pendik, Istanbul, Turkey; 4 Bezmialem Vakif University, Department of Pediatric Infectious Diseases, Istanbul, Turkey; 5 Boston Childrens Hospital, Department of Pediatric/Urgent Care, USA; 6 Istanbul University, Istanbul Faculty of Medicine, Department of Ophthalmology, Pendik, Istanbul, Turkey; 7 Marmara University Medical Faculty, Department of Microbiology, Istanbul, Turkey

**Keywords:** Fungal infection, paecilomyces, voriconazole

## Abstract

Fungal infections like *Paecilomyces keratitis* have emerged in childhood recently. The diagnosis and treatment of *Paecilomyces keratitis* is difficult and the outcome is usually poor. Corneal culture should be performed on fungal media such as Sabouraud glucose neopeptone agar (SDA) as soon as possible for diagnosis. We report a rare case of *Paecilomyces keratitis* in an immunocompetent child, which was unresponsive to amphotericin B. The case was managed by a multidisciplinary approach involving the departments of ophthalmology, microbiology and pediatric infectious diseases. We want to draw attention once again that fungal keratitis caused by unusual agents are increasing. Physicians should consider fungal causes of keratitis, in patients with some predisposing factors like ocular surgery and prolonged use of topical corticosteroids.

## Introduction

*Paecilomyces species* are saprobic filamentous fungi and mainly cause ocular infections that frequently followed intraocular lens implantation, trauma and ocular surgery [1]. This fungus can be found in air, soil, wood and decaying vegetation. It is a rare but potentially serious pathogen that causes of filamentous fungal keratitis in children. *Paecilomyces species* are resistant to many antifungal agents, hence *Paecilomyces keratitis* treatment is frequently difficult and usually results poor outcome [2]. We report an unusual pediatric case of postoperative Paecilomyces keratitis that required vigorous antifungal treatment-systemic, topical and intracameral, and healed with a vascularized scar. We want to emphasize that corneal sample should performed on fungal media such as Sabouraud glucose neopeptone agar (SDA) as soon as possible in suspected fungal cases. Case management is important for prevent further complications.

## Patient and observation

**Figure 1:**
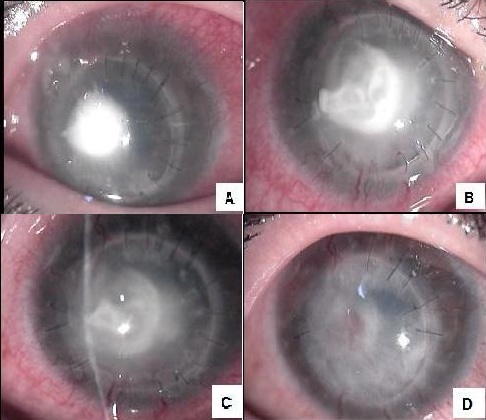
On slit lamp examination (right eye): A) the central corneal abscess with corneal edema and hypopyon on admission; B) healing with vascularized scar on cornea after voriconazole and terbinafine treatment at day 4; C) day 8; D) day 15

**Figure 2:**
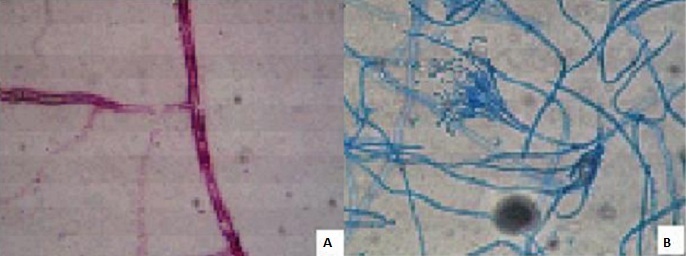
A) microscopic examination of corneal scrapings revealed a septate and branching fungal hyphae; B) branching conidiophores arising from infrequent septate hyphae with tapering phialides and chains of conidia characterise *Paecilomyces spp* (Lactophenol cotton blue stain)

A-14-year-old boy with a history of acute hydrops due to keratoconus in the right eye was treated by keratoplasty. Prednisolone acetate 1% and ciprofloxacin 0.3% eye drops were given postoperatively. On the 30th day after the surgery he complained of severe pain, redness and decreasing vision in the right eye and admitted to hospital. On slit lamp examination, there was a 3x3 mm central abscess on the graft involving all layers of the cornea in the right eye; the left eye examination was normal (Figure 1 A). Corneal scraping was performed and material was obtained for smears and cultures. Corticosteroid eye drop was discontinued and broad-spectrum topical antibiotic therapy with fortified vancomycine (50 mg/ml) and gentamicine (14 mg/ml) was initiated. Treatment was subsequently switched to topical (2%, 6 times a day) and systemic (6 mg/kg intravenous, once a day) fluconazole and topical (0.3%, 6 times a day) amphotericin B since microscopic examination of the corneal scrapings revealed septate branching fungal hyphal structures (Figure 2 A). Amphotericin B was also injected intracamerally at a dosage of 0.005 mg/0.1mL. After 1 week of treatment, the clinical picture deteriorated with elevation of the abscess and impending perforation. The viral and bacterial investigations were negative. In the meantime, mold colonies appeared on SDA (first week of incubation). On examination of fungal culture, the colony was flat, powdery, velvety and yellowish in the obverse side and white to pinkish color in the reverse side. The fungal isolate was identified as a *Paecilomyces species* by the microbiology laboratory (Figure 2 B). Antifungal therapy was modified to oral terbinafine (250 mg once a day) plus topical (1%, 6 times a day) and systemic (6 mg/kg twice on day 1 and 4 mg/kg twice on the following days, intravenous) voriconazole. Repeat fungal cultures were negative. Clinical improvement was observed and the corneal lesion healed with the vascularized scar tissue (Figure 1 B). Terbinafine and voriconazole were discontinued after, respectively (Figure 1 C, D).

## Discussion

Fungal infections are important cause of morbidity and mortality in childhood. More than 70 genera of filamentous fungi and yeasts have been reported to cause fungal keratitis. The reports of fungal ocular infections related with the impared host defense mechanisms have been increased in recent years [3]. Ocular surgery and prolonged use of topical corticosteroids were stated to be the major risk factors associated with fungal keratitis. *Paecilomyces species* are saprobic, filamentous fungi which belong to hyphomycetes class. All hyaline hyphomycetes, including species of *Aspergillus*, *Scedosporium*, *Fusarium*, *Acremonium* and *Paecilomyces*, exhibit similar appearances in clinical specimens on microscopic examination of the infected tissue. Their septate, nonpigmented acute branching (about 45° angles) hyphae render them indistinguishable from each other on microscopic examination. Correlation with culture results and macroscopic morphology is needed for definitive identification at least at the genus level. Once isolated, molds are primarily identified according to their macroscopic morphology on culture media and type of reproductive structures observed microscopically [4]. *Paecilomyces species* usually cause keratitis, endocarditis, sinusitis, fungemia, pulmonary, cutaneous and subcutaneous infections in an immunocompromised host and similar infections in an immunocompetent host are very rare.

*Paecilomyces* infections have been reported in patients with or without predisposing factors. About 100 cases of these infections have been reported related to the surgical procedure or immunocompromised hosts [5]. Although *Paecilomyces keratitis* caused by lilacinus strain is more common, other Paecilomyces strains such as farinosus, marquandii, variotti and viridis keratitis were reported in the literature [6]. A review study showed that 31% of 42 *Paecilomyces keratitis* cases were associated with chronic keratopathy or previous ocular surgery, 26% appeared following a corneal trauma and 24% occurred in soft contact lens wearers. Other causes were endogenous endophthalmitis and infectious scleritis [7]. Fungal cultures should be obtained as early as possible for diagnosis. Correct identification for fungal pathogen is important for appropriate treatment and to prevent further complications. Antifungal susceptibility tests are not performed routinely for saprobic molds because of difficulties in standardization and reading of the end points.

This case was managed by a multidisciplinary approach involving the departments of ophthalmology, microbiology and pediatric infectious diseases. Experience in the treatment of keratitis due to *Paecilomyces species* is limited and the optimal antifungal treatment remains unknown. There has been a trend toward using voriconazole, which has the broadest spectrum of the azole antifungals, for the treatment of fungal keratitis. Although *P. Lilacinus keratitis* is resistant to many antifungal agents, voriconazole has been found effective for treatment. Voriconazole has an excellent topical and oral bioavailability and therapeutic aqueous and vitreous levels are achieved after the topical and oral administration of voriconazole [8, 9]. Terbinafine belongs to the allylamine group of antifungals and inhibits squalene epoxidase, an enzyme involved in sterol metabolism of the cell membrane. Terbinafine and voriconazole combination was synergistic against all tested strains of *Paecilomyces* [10]. We combined terbinafine with voriconazole to treat and prevent further complications. Anderson et al. also reported a case of *Paecilomyces keratitis* associated with a retained intracorneal hair that was resistant to routine antifungal agents but was successfully treated with terbinafine and voriconazole combination therapy [3]. Although a treatment algorithm for *Paecilomyces keratitis* has not been defined, it may be described with additional experiences in the future.

## Conclusion

We want to draw attention once again that fungal keratitis caused by unusual agents are increasing. Physicians should consider fungal causes of keratitis, in patients with some predisposing factors like ocular surgery and prolonged use of topical corticosteroids. We also suggest that corneal cultures should be performed. Voriconazole was effective in treatment for keratitis caused by *Paecilomyces species*.
